# Comparison of explicit values clarification method (VCM), implicit VCM and no VCM decision aids for men considering prostate cancer screening: protocol of a randomized trial

**DOI:** 10.1186/s12911-020-1094-3

**Published:** 2020-04-29

**Authors:** S. Baptista, B. Heleno, A. Teixeira, K. L. Taylor, C. Martins

**Affiliations:** 1 Department of Community Medicine, Information and Health Decision Sciences (MEDCIDS), Faculty of Medicine, University of Porto, Al. Prof. Hernâni Monteiro, 4200 - 319 Porto, Portugal; 2 Serpa Pinto Family Medicine Unit, Agrupamento de Centros de Saúde Porto Ocidental, Porto, Portugal; 3 Centre for Health Technology and Services Research (CINTESIS), University of Porto, Porto, Portugal; 4 Comprehensive Health Research Center (CHRC), Universidade NOVA de Lisboa, Lisboa, Portugal.5 - NOVA Medical School|Faculdade de Ciências Médicas, Universidade NOVA de Lisboa, Lisbon, Portugal; 5 Department of Oncology, Lombardi Comprehensive Cancer Center, Georgetown University, Washington, D.C, United States of America

**Keywords:** Shared decision-making, Decision aid, Screening, Prostate cancer, Values clarification, Decisional conflict

## Abstract

**Background:**

Screening with prostate-specific antigen (PSA) test for prostate cancer is considered a preference sensitive decision; meaning it does not only depend on what is best from a medical point of view, but also from a patient value standpoint. Decision aids are evidence-based tools which are shown to help people feel clearer about their values; therefore it has been advocated that decision aids should contain a specific values clarification method (VCM). VCMs may be either implicit or explicit, but the evidence concerning the best method is scarce. We aim to compare the perceived clarity of personal values in men considering PSA screening using decision aids with no VCM versus an implicit VCM versus an explicit VCM.

**Methods:**

Male factory employees from an industrial facility in the Northern region of Portugal aged 50 to 69 years old will be randomly assigned to one of three decision aid groups used to support prostate cancer screening decisions: (i) decision aid with information only (control), (ii) decision aid with information plus an implicit VCM, (iii) decision aid with information plus an explicit VCM.

Men will be allowed release time from work to attend a session at their workplace. After a brief oral presentation, those willing to participate in the study will fill the baseline questionnaire, plus a 5 point-Likert scale question about intentions to undergo screening, and will then receive the intervention materials to complete.

We estimated a total sample size of 276 participants; with 92 in each group.

The primary outcome will be the perceived clarity of personal values assessed by the Portuguese validated translation of the three subscales of the Decisional Conflict Scale. Secondary outcomes will be intention to be screened (before and after the intervention), the total score from the Decisional Conflict Scale and the self-report of having or not undergone screening at 6 months.

**Discussion:**

This study will add to the body of evidence on the role of decision aids to support health preference-sensitive choices and provide further insight on the impact of different methods for eliciting people’s values embedded within a decision aid.

**Trial registration:**

NCT03988673 - clinicalTrials.gov (2019/06/17).

## Background

Prostate cancer is a leading cause of men cancer worldwide [1]. Nevertheless, screening for prostate cancer using a prostate specific antigen (PSA) test remains a controversial issue; implying a trade-off between benefits (mortality reduction, early diagnosis) and risks (high instances of overdiagnosis and overtreatment in conjunction with the consequent side effects, as well as false positive and false negative test results) [2, 3]. Thus, the PSA test used to screen for prostate cancer is considered a preference sensitive decision; meaning it does not only depend on what is best from a medical point of view, but also taking into account patient preferences; i.e., the values a patient attaches to the advantages and disadvantages of that option [4].

According to the International Patient Decision Aids Standards (IPDAS) Collaboration, decision aids are evidence-based tools designed to help people participate in decision making about health care options with the aim of improving the quality of the decision [5]. The most recent systematic review concluded that decision aids could increase patient knowledge about screening, make people feel clearer about their values, reduce decisional conflict, and promote an active patient role in decision making [6].

In consideration of evidence on the role of decision aids in helping to clarify patient values, the IPDAS suggested that decision aids should include some values clarification methods (VCMs) [7]. VCMs are defined as strategies designed to help patients evaluate the desirability and attributes of different options in order to identify the option they prefer [8]. The methods used for values clarification are often classified as being either implicit or explicit [9]. With implicit techniques, patients are presented the pros and cons of the available options, on a balance sheet, for instance, and are expected to weigh up the desirability of the different options on their own and, ultimately, they will develop a preference for one of the options [10]. On the other hand, explicit VCMs are designed to engage the patient in tasks to specifically compare the relative importance of characteristics relevant to a decision [11]. Examples of such explicit methods are rating and ranking tasks as well as social matching which consists of viewing others engaged in decision making and identifying one’s own values to the values of these individuals [12].

In a previous version of the Cochrane review of patient decision aids, 59.1% of the decision aids considered included explicit methods used to clarify values. Decision aids with explicit VCMs resulted in a higher proportion of patients choosing a screening option congruent with their values (RR = 1.51; 95% CI 1.17 to1.96; *n* = 13) in comparison to a simple decision aid without explicit values clarification or usual care [13]. Implicit VCMs were not assessed in this review.

Nevertheless, there is still scarce evidence concerning whether implicit and explicit values clarification methods may make a difference on patient reported values and preferences compared to instance where no VCMs are used.

While engaging in an explicit values clarification task, the patient may gain insight into the value he/she assigns to each option, which could help him/her to select a preferred option and to be able to communicate his/her preferences to others [10]. According to this rationale, values clarification methods may be more helpful when, in addition to helping users to realise what matters to them, they clarify how what matters to them determines which option may be the preferred one [14]. On the other hand, explicitly exploring the pros and cons of each option may increase uncertainty towards the choice and, thus, implicit VCMs may be superior [9].

### Objectives

To compare the perceived clarity of personal values in men considering PSA screening using decision aids with no VCMs versus an implicit VCM versus an explicit VCM.

### Trial design

Parallel three group (1:1:1) randomized controlled trial.

## Methods

**Fig. 1:**
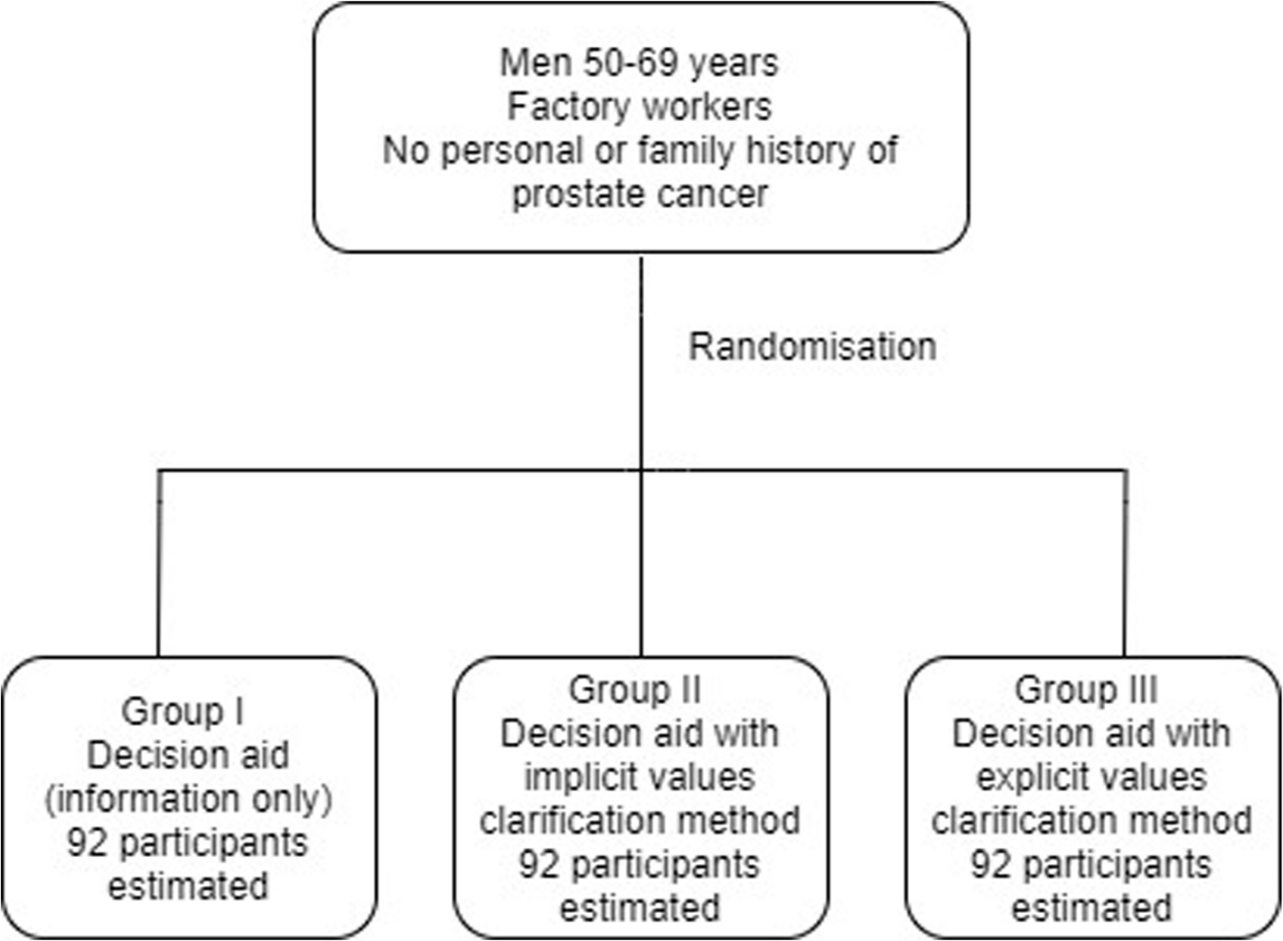
Randomisation of trial participants

**Fig. 2:**
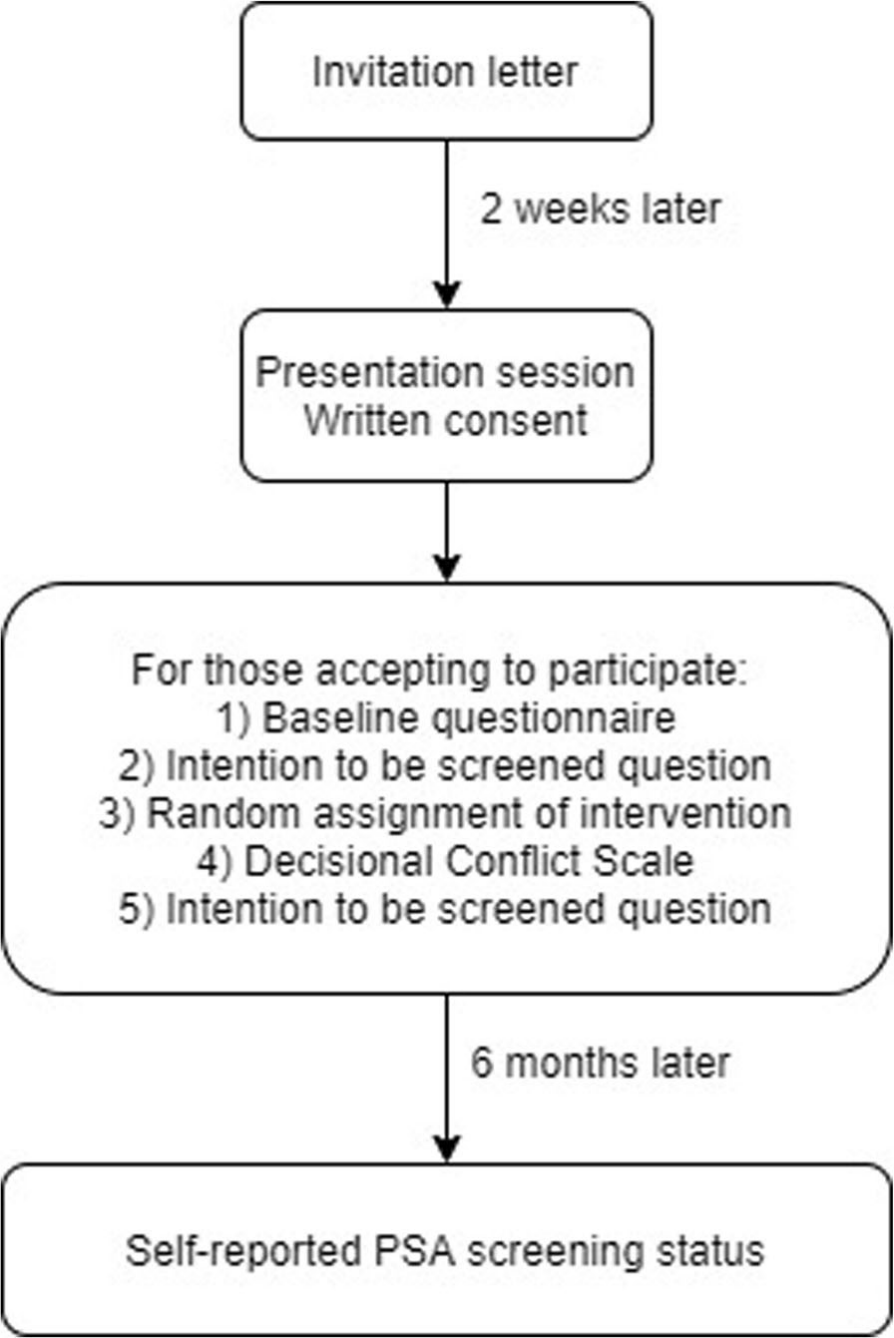
Sequence of main trial steps

50 to 69-year-old male factory workers will be randomly assigned to one of three decision aid groups supporting prostate cancer screening decisions: (i) decision aid with information only (Control), (ii) decision aid with information plus an implicit VCM, and (iii) decision aid with information plus an explicit VCM. Figs 1 and 2 and Table 1 summarize the main trial steps.

**Table 1:** Schedule of enrolment, interventions, and assessments

	STUDY PERIOD					
	Enrolment	Allocation	Post-allocation			Close-out
TIMEPOINT**	***-t***_***1***_ **2 weeks before**	**0** **session**	***t***_***1***_ **session**	***t***_***2***_ **session**	***t***_***3***_ **session**	***t***_***4***_ **6 months later**
**ENROLMENT:**						
**Eligibility screen**	X					
**Informed consent**	X					
**Allocation**		X				
**INTERVENTIONS:**						
***[Decision aid without VCM]***				X		
***[Decision aid with implicit VCM]***				X		
***[Decision aid with explicit VCM]***				X		
**ASSESSMENTS:**						
***[baseline variables]***			X			
***[Screening intention]***			X		X	
***[Decisional conflict scale]***					X	
***[Self-reported PSA screening]***						X

### Study setting

The trial will be held at an industrial facility in the Northern region of Portugal.

### Eligibility criteria

To participate in the study, men should be aged 50 to 69 years, be at average risk for prostate cancer (e.g., no personal or family history of prostate cancer; thus, not at high risk), and be able to provide written informed consent. Those unable to understand written Portuguese will be excluded. The baseline questionnaire will assess the participants’ eligibility.

### Interventions

“Making the best choice” is an English language prostate cancer printed screening decision aid which has been rigorously developed [15] and extensively tested [16–19] by a workgroup from Georgetown University (United States of America). We were given permission to translate and adapt the decision aid to the Portuguese population. The decision aid includes different sections: introduction to prostate and prostate cancer; screening tests (results, benefits and risks); treatment (options, benefits and risks); prostate cancer risk factors; encouragement to share decision-making with a physician; a values clarification method; and resources to access additional information (references and a glossary). KT, who was the principal investigator of the original decision aid is a co-investigator in this project. The results from the translation and cultural adaptation of the decision aid to the Portuguese population were recently published [20]. The testimonials section of the decision aid will be excluded in this study since this can be considered a type of values clarification method and could thus bias the results.

The VCM in the decision aid was adapted from a previous study [21] and is composed of 10 statements addressing the most important aspects involved in prostate cancer screening decisions. The control group will receive the decision aid without any VCMs. One intervention group will receive the decision aid with the implicit VCM. In this version of the decision aid, men will be presented with 10 statements. For the first set of five statements, participants will be informed that “Men who have made these statements often decide to get screened” and for the second set of five statements, they will be informed that “Men who have made these statements often decide NOT to get screened.” The other intervention group will receive the decision aid with an explicit VCM. For each of the 10 statements, men will be asked whether each sentence sounds like him or not.

Men will be asked their personal position towards screening before and after reading the decision aid.

### Outcomes

The primary outcome will be the perceived clarity of personal values assessed by the Portuguese validated translation of the three subscales of the Decisional Conflict Scale (DCS) [22]. The Values Clarity subscores range from 0 (feels extremely clear about personal values for benefits and risks/side effects) to 100 (feels extremely unclear about personal values); lower scores suggest better clarity.

Secondary outcomes will be intention to be screened and the total score from DCS. Respondents will be asked a single question about their intention to undergo a PSA screening, using a 5 point-Likert scale (ranging from “strongly disagree” to “strongly agree”; intention to undergo PSA screening will be considered positive if the respondent replies with “agree” or “strongly agree”). Total scores from the 16-questions DCS (including the subscales values clarity, informed decision making, effective decision making, decision making support, decision making uncertainty) range from 0 (no decisional conflict) to 100 (extremely high decisional conflict), discriminating between those who make and delay decisions [23]. Furthermore, prostate cancer screening status at 6 months after the intervention will be assessed by way of a self-report.

### Patient timeline

After a brief oral presentation session, men willing to participate in the study will be invited to stay and fill the baseline questionnaire plus a 5 point-Likert scale question about intention to undergo screening. These will have the same numeric code as the sealed envelopes containing the materials to be distributed. Participants should then complete the required questions after intervention assignment during the same session.

### Sample size

We estimated a total sample size of 276 participants, 92 in each group, using the ANOVA test for three groups and considering an average effect size of 0.2 with a significance level of 0.05 and 80% power, attending to the psychometric properties of the DCS and also accounting for 10% of participants leaving the study before conclusion.

### Recruitment

Two weeks in advance, male employees will be sent an invitation letter to participate in a facultative session about prostate cancer screening and presenting the trial and how to participate. The session will be held at their workplace, during business hours, and will be presented by the lead investigator. During the session, men will be invited to stay after the initial presentation if they are willing to participate in the trial. They will then be presented with written information about the project, the lead investigator will be available to answer any questions and the willing participants will be asked to give their written consent. Men will be allowed release-time from work to participate in the session.

### Assignment of interventions, sequence generation and blinding

To ensure allocation concealment, a computer-generated random schedule (unstratified randomization with a 1:1:1 allocation ratio) will be generated by a statistician not otherwise involved in the trial. Blocking will be reported in an appendix, even though all participants will be randomized at the same time. The same statistician will prepare closed, equally sized, sequentially numbered, opaque, sealed white envelopes. The set of envelopes will be given to the lead investigator which will then distribute the envelopes to the participating men.

### Blinding

Due to the nature of the intervention, it will be impossible to fully blind participants. In order to minimize bias, participants will know that different formats of the decision aid will be assessed, but they will not be informed about the differences in formats or about the research hypotheses. After the decision aid packages are given to participants, the lead investigator will no longer interact with participants; thus, she will not be aware of which decision aid has been allocated to individual participants. Data collection forms will be collected by other members of the research team. Since the main outcome is self-reported, it is impossible to blind outcome assessors; however, data collection procedures will be the same, regardless of the allocated group. The statistician assessing the data and the first author will not be blinded to allocation when analyzing or drafting the manuscript, respectively.

### Data collection methods

Baseline demographic data will be collected by way of pre-intervention questionnaires. Participants will also answer a 5-point Likert-scale question about intention to be screened with PSA test.

Upon receiving the decision aid and, when applicable, values clarification method reading/completion, participants will answer the post-intervention questionnaire, containing the Portuguese validated translation of the DCS, as well as the same above mentioned Likert-scale question about intention to undergo PSA screening.

### Data management

Participant paper files, only identifiable by a participant ID number, will be stored in numerical order in a secure place at the University of Porto. Participant paper files will be maintained in storage for a period of 3 years after completion of the study. All anonymized data will be entered electronically and, only after all data has been entered, will researchers contact the statistician who prepared the randomization schedule to know which group was assigned to individual participants access to the study data will be restricted to the research team based at University of Porto. A data tracking system with time stamps will be implemented for audit purposes. A password system will be utilized to control access to de-identified electronic data and these passwords will be changed every 3 months.

To ensure that we can follow participants for screening participation after 12 months, we will keep a single file which links participant ID number to participant name, phone number and one next of kin phone number. This electronic file will be kept by a team researcher not based at the University of Porto.

Anonymized data will be analyzed on a personal computer belonging to one of the authors based at the University of Porto.

### Statistical methods

After collection, data will be analyzed using Microsoft Excel 2016® and SPSS Statistics 25.0®. Participants will be analyzed in the group they were randomized to, regardless of actual intervention received. In order to analyze between group differences concerning perceived clarity of personal values, we will use One-way ANOVA (if the variable distribution is normal) or Kruskal-Wallis (if the variables are not distributed normally). Normal distribution of variables will be assessed by observing the respective histograms and validated with the Kolmogorov-Smirnov test. For continuous variables with normal distributions, data will be described using mean (M), standard deviation (SD), minimum (min), and maximum (max). For continuous variables not distributed normally, data will be described using median (Med) and the respective interquartile range [Q_1_; Q_3_]; Q_1_ being the first quartile and Q_3_ being the third quartile.

If either the ANOVA or Kruskal-Wallis tests demonstrates significant differences between groups, multiple comparisons will be conducted between groups pairs in order to detect between which groups there are differences.

To test independence between categorical variables, we will use Chi-square test for independence. Categorical variables will be described using absolute and relative frequencies, N (%). *p*-values ≤0.05 will be considered significant.

DCS total score is composed of 16 items (each one with a 5-point Likert response scale) divided in five subscores: uncertainty subscore (3 items), informed subscore (3 items), values clarity subscore (3 items), support subscore (3 items), effective decision subscore (4 items). For each participant partial scores and a total score will be calculated. Concerning missing values, those participants who do not answer at least 50% of the items of each subscale will be excluded from the analysis. The total score will be presented on a 0–100 scale [23].

If there are baseline differences between groups, we will perform multivariate analysis using linear multiple regression.

### Data monitoring

Given the expected safety during the trial, no data monitoring committee will be established or interim analyses conducted.

### Protocol amendments

In the case of substantive protocol amendments an addendum will be submitted both to Ethics Committee and the Trial registry.

### Consent

After adequate explanation on the study procedures and aims by workgroup researchers and having the opportunity to discuss any questions or doubts with them, participants will fill in a written consent form, if they are willing to participate in the trial.

### Confidentiality

All the information related to the study will be stored securely at the study site. All participant information will be stored in locked file cabinets in areas with limited access at the University of Porto. All records containing identity elements, such as informed consent forms, will be stored elsewhere, separate from study records, and identifiable by a code number. All local databases will be secured with password. Any document with information that potentially links participant ID numbers to other identifying information will be stored in a separate, locked file in an area with limited access.

### Dissemination policy

Results from this study will be disseminated in peer-reviewed publications, conference presentations, reports, and in a PhD thesis. There will be no publication restrictions. Authors will comply with ICMJE authorship criteria. No professional writers will be used. The trial protocol will be published in an open access journal.

## Discussion

To our knowledge, this will be the first randomized controlled trial comparing information only (no VMC) versus an implicit VCM versus an explicit VCM embedded within a full decision aid to support the decisions of men regarding prostate cancer screening with PSA.

This study will add to the body of evidence on the role of decision aids to support health preference-sensitive choices and provide further insight on the impact of different methods for eliciting people’s values embedded within a decision aid.

By assessing those men who underwent PSA screening 6 months after the intervention, we will be able to evaluate the effect of the different VCMs on the actual decision.

Conducting our research at an industrial workplace rather than a clinical setting may be an opportunity to reach less educated populations not usually included in trials and which could probably benefit more from the support of decision aids in decision-making. On the other hand, this may also represent a limitation from our study since the findings may not be generalizable to other male populations. Contamination may be another issue, particularly concerning the report of PSA test at 6 months, as men’s decision can be attributed to a wide range of factors and not only those strictly related to the study. Therefore, results should be interpreted with caution.

Future works should focus on deepening our understanding on the role of different VCM methods within decision aids in helping to clarify patient values and use more distal outcomes.

## Data Availability

The datasets used during the current study will be available from the corresponding author on reasonable request.

## Abbreviations

DCS: Decisional Conflict Scale
IPDAS: International Patient Decision Aids Standards Collaboration
PSA: Prostate specific antigen
VCM: values clarification method
